# A New Sensitive Sensor Test for Capturing and Evaluating Bacteria and Viruses in Airborne Aerosols

**DOI:** 10.3390/s25133866

**Published:** 2025-06-21

**Authors:** Roman Pernica, Zoltán Szabó, Martin Čáp, Oto Pavliš, Pavla Kubíčková, Jiri Zukal, Pavel Fiala

**Affiliations:** 1 Department of Theoretical and Experimental Electrical Engineering, Brno University of Technology, Technická 12, 616 00 Brno, Czech Republic; 15788@vut.cz (R.P.); szaboz@vut.cz (Z.S.);; 2 Military Health Institute, Biological Defence Department, 56166 Techonin, Czech Republic; oto.pavlis@email.cz (O.P.); pafcule@centrum.cz (P.K.)

**Keywords:** sensor, PCR, aerosol, COVID-19, virus, bacteria, detection, electromagnetic field

## Abstract

**Highlights:**

Why can’t viruses be captured by the currently known bacteria detection devices? This question can be answered by the uniqueness of the electromagnetic properties of microorganisms and organic nanoparticles. The basic parameter for capturing these substances, the electric field, is the interaction with the electric field of the sensor system, the electric charge of the substance. This affects the development of methods and metrology, the results and quality of capture measurements and the evaluation of tested substances.

**Abstract:**

In this paper, the authors describe an electromagnetic–hydrodynamic (EMHD) model of the airborne microbiological agent detection concept for the design of a sensor to identify the presence of airborne bacteria and viruses. Based on the model and a laboratory test, a methodology was proposed for the capture and subsequent detection of low-concentration bacterial and viral agents in airborne aerosols. A physical–biological approach was proposed to detect microorganisms based on their physical properties. The principle was validated in the laboratory on samples of defined concentrated water aerosols of Bacillus subtilis (BS) and feline infectious peritonitis virus (FIVP). Repeated tests with different concentrations were performed in the laboratory conditions.

## 1. Introduction

Between 2020 and 2023, it became a critical scientific objective to develop reliable and reproducible detection methods for evaluating the presence of hazardous microorganisms in airborne aerosols. This period was defined by the COVID-19 pandemic. Initial testing showed that previously marketed mobile bacterial collection systems were unable to effectively detect relatively small organic particles—viruses. During this time, monitoring of the target pathogen, the SARS-CoV-2 coronavirus, was required. Devices such as the BioCapture 650, FLIR IBAC 2, FLIR IBAC OEM, model 1329 OEM, and others failed to meet the necessary parameters for sampling aerosolized air and detecting the virus. Research was initiated and carried out by assembled teams. The goal was to identify methods and protocols for reliable and repeatable capture of target agents at low concentrations in air aerosols. Known methods included surface swabbing and subsequent analysis, such as PCR techniques.

The project “System for sampling and detection of coronavirus and other respiratory pathogens from the air” has been proposed and approved for the period 2020 to 2023. It was represented by scientists from the fields of microbiology, biochemistry, the Ministry of Defence and theoretical electrical engineering.

This project dealt with the research and development of equipment capable of sampling microorganisms from airborne aerosols, specifically airborne viruses. The sampling device was designed to capture pathogens presented in the air using sampling matrices, which are further assessed using molecular biology methods, such as quantitative PCR. The main purpose of a sampler is to monitor areas of high concentration and movement of people. This work was based on well-known [1] or concurrently published papers [2,3,4,5,6,7,8,9], which provided insights for the range of parameters of the monitored agents and from a multidisciplinary perspective.

The output of this research was functional samples of devices capable of detecting and subsequently analyzing in-air aerosol from the guaranteed sensor surroundings of objects of organic origin of micro- and nano-size orders. The devices were designed to capture objects on fabric filter arrays, on the surface of gels, and on the surfaces of plasma carriers for further evaluation using real-time PCR (qPCR) or reverse transcription real-time PCR (RT-qPCR) devices. Non-pathogenic coronavirus representatives were evaluated in the laboratory and in real conditions outside the laboratory.

Preliminary research was conducted in the form of surface swabs and the evaluation of SARS-CoV-2 presence in public areas such as supermarkets and post offices. The swabs were analyzed using PCR. Tests of available mobile detection and sampling devices for bacterial collection in open spaces did not provide satisfactory results for SARS-CoV-2 detection. Fundamental research into new pathogen monitoring approaches was launched. A test chamber for a biosafety level 4 laboratory was designed. Conditions for aerosol formation and distribution with adjustable pathogen concentrations were established. Tests with selected microorganisms began, including the feline infectious peritonitis virus (FIPV), strain WSU 79-1146, ATCC: VR-990, which was prepared in a concentrated form and introduced into the test chamber using a fogger. It was found that due to electric charges on the chamber walls and transport pipes, FIPV could not be detected via swabbing or PCR in areas beyond the fogger. Thus, the research shifted to exploring the influence of electromagnetic fields on FIPV.

Initial pathogen detection tests in aerosols, without considering the electric charge of the agent and environment, failed to enable capture and detection. Repeated tests were carried out in BSL-4 safety labs. Aerosol containing a known concentration of agents was injected into a specially constructed plastic chamber. Swabs and PCR tests were performed at various locations along the route from the fogger to the impinger. At a distance of 10 cm from the fogger, the result was negative. A non-zero electric charge was detected on the plastic components of the box and aerosol delivery pipes. Without appropriate electric field settings, the agent did not disperse from the fogger to the chamber. When proper electrostatic conditions were applied, the aerosol containing the agent reached the chamber, and its presence was detected using PCR.

A detector concept was proposed as a device that captures the pathogen and subsequently allows its evaluation using PCR.

The sensor is not designed to provide an immediate electrical signal output corresponding to specific pathogenic microorganisms. Rather, it is a device optimized to capture agents onto a suitable matrix, which is then microbiologically processed, and the amount and type of agents are detected via PCR.

## 2. Design of Object Sensing Concept—Electromagnetic Separation

**Figure 1:**
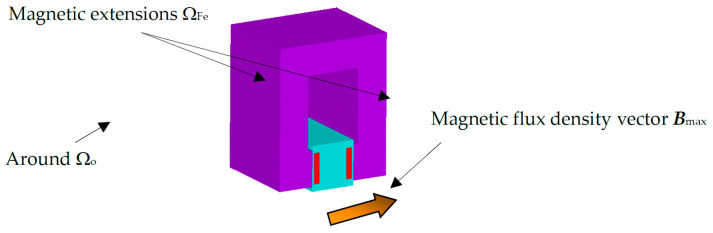
Principal arrangement of the classical concept of the Wien filter—magnetic circuit.

The works [9,10,11,12,13,14,15,16,17,18,19] suggest the use and application of several non-standard options for object detection based on the properties of biological nano-objects and their detection in airborne aerosols [2,3,4,5,6,7,8,9]. The first approach to the detection of biological objects (viruses, bacteria) is based on the findings published in papers [2,3,4,5,6,7,8,9] and is based on the use of electromagnetic properties [10,11,12] of agents in the dimensional categories of nano-objects and subnano-objects. Such a solution is based on the separation of agents based on Wien filter principles (Figure 1). The basic concept/design of the Wien filter space for the proposed purposes is shown in Figure 1.

**Figure 2:**
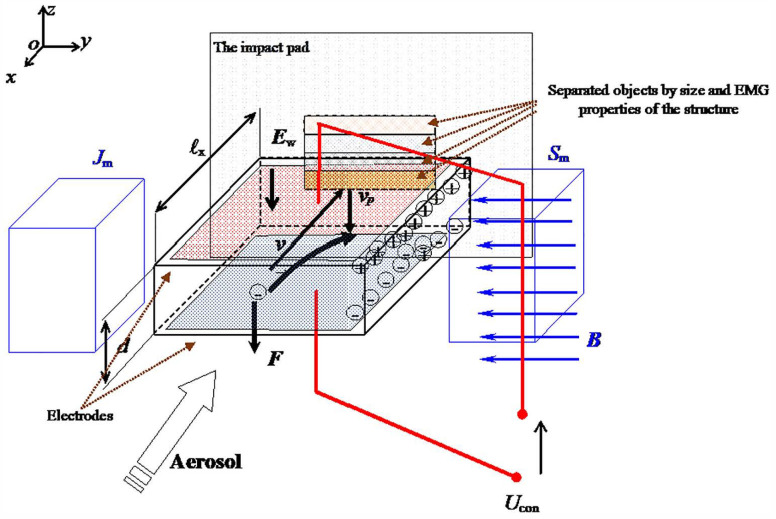
The principle of operation of the proposed Wien filter concept for the separation of micro-/nano-biological objects.

The parameters for the first theoretical approximation of the design of an agent splitter that would spatially separate the contained subnano-, nano-, and microparticles consist of the following properties: The estimated content bacteria/viruses, number *N*_sum_ = 1·10^9^, with mass *m*_sum_ = 1.2 kg, then leads to the order of the mass of one bacterium/virus *m*_b_ = 0.12 µg, the object uptake rate from airborne aerosol *v* = 0.1–2 m/s. The velocity without deflection of the object (bacteria/virus) is given by

(1) $$v=\frac{E}{B},$$

where *E* is the electric field strength in the Wien filter region and *B* is the specific magnetic flux in the filter. From this relation, an estimate of the possible orders of magnitude of the setup for the separation of agents can be found:

(2) v=1 m/sE=0.5VmB=0.5T,

Then the concept of the separator can be schematically captured and illustrated in Figure 2. Based on fundamental knowledge of electromagnetic fields (e.g., as published in [11,14]), it is possible to determine the force ***F*** acting on parts of a nano-object within the defined sensing area. This enables setting limits on the intensity of the electric field E to avoid DNA degradation. Based on this, threshold electric potentials and magnetic flux densities ***B*** were established to ensure compliance with this requirement.

**Figure 3:**
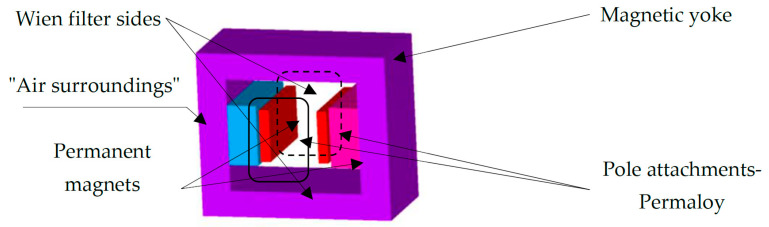
Geometric model—Wien filter with permanent magnets.

A numerical model of the filter was designed and built (Figure 1 and Figure 3). The first tests with microparticles and dust up to 1 μm were carried out. As an alternative to the Wien filter, a permanent magnet filter model was designed for a solution not requiring a demanding electrical supply, the supply being only the electrostatic arrangement [13,14,15,16,17,18,19,20] of the filter section (Figure 3).

**Figure 4:**
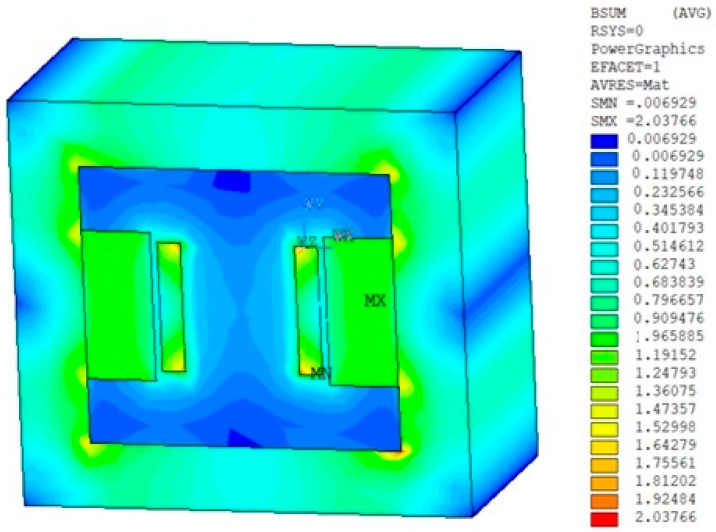
Magnetic flux density distribution *B* Wien filter model.

From the models and their analyses using the finite element method [20,21,22], it is clear that a magnetic circuit can be constructed using permanent magnets. Therefore, the first concept design was modified into the shape shown in Figure 3. Subsequently, the analysis and evaluation from the proposed geometrical model were carried out; the distribution of magnetic induction *B* (Figure 4) was found, as well as the relative permeability *μ*_r_ and other EMG field quantities. The permanent magnet FeNeB was considered, *H*_c_ = 850 kA/m, *B*_r_ = 1.2 T.

**Model virus and bacteria preparation**

Feline infectious peritonitis virus; strain WSU 79-1146; ATCC: VR-990 (FIPV) was multiplied on Crandell–Rees feline kidney cells in a total volume of 50 mm. Bacillus subtilis spores were prepared by scraping colonies from blood agar containing 4% sheep blood after 24 h of culture. Colonies were resuspended in PBS and diluted to OD600 = 1.3–1.5, which corresponds to approximately 10^8^ bacterial cells per mL.

**Design of the testing cabinet**

**Figure 5:**
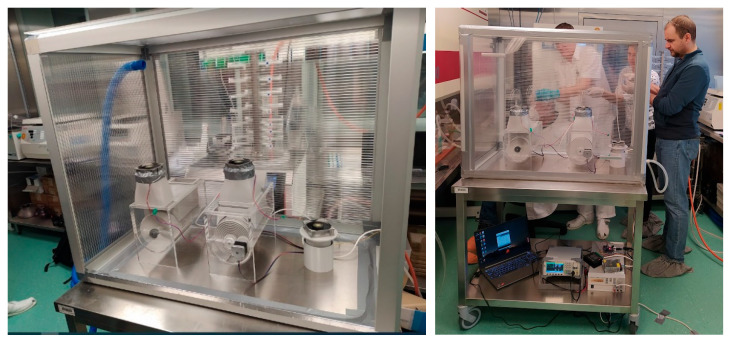
The cabinet for air aerosol sampling with agents.

The construction of the enclosure for testing biological agents in air aerosols was constructed from anodized aluminum L-sections and filled with polycarbonate (Figure 5) so that the test area was hermetically sealed from the surrounding environment and the operator.

When setting the electric field intensity E, the limit for FIPV was respected, with *E*_maxFIPV_ = 800 V/m.

**Figure 6:**
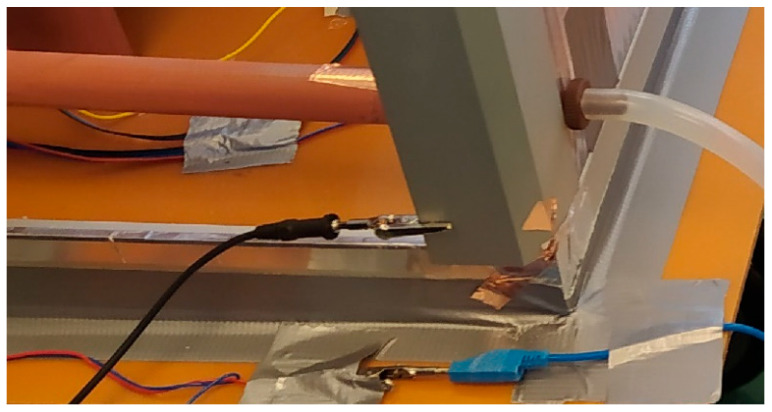
The cabinet for air aerosol sampling with agents; contact to reference electric potential.

**Figure 7:**
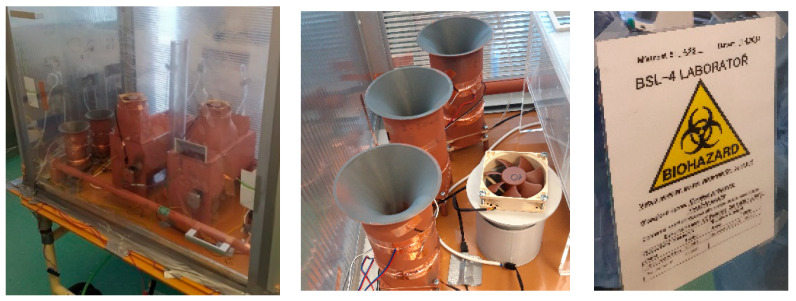
The cabinet for air aerosol sampling with agents; airtight closure.

The polycarbonate was coated with foil to minimize electrical charge on the wall surfaces. The entire enclosure was connected to a single reference potential in the next step (Figure 6). The enclosure was sealed with a front wall that was hermetically sealed prior to testing (Figure 7). In the test box with safe separation from the external environment, the methods were tested using the prepared equipment [15,17,22]. Aerosol was generated from the bottom of the box using a vertical glass tube. An example of the placement and testing of the designed sensors with the Wien filter [20] is shown in Figure 7.

The magnetohydrodynamic principle [20] of separation and sensing of biological objects with the application of the Wien filter was tested together with other known principles [22,23,24,25]. The description of the basic parameters of the compared devices using the principles of the tested capture systems and devices can be characterized in the following parameters [26]. This paper further focuses only on the electrohydrodynamic principle of agent separation.

**Figure 8:**
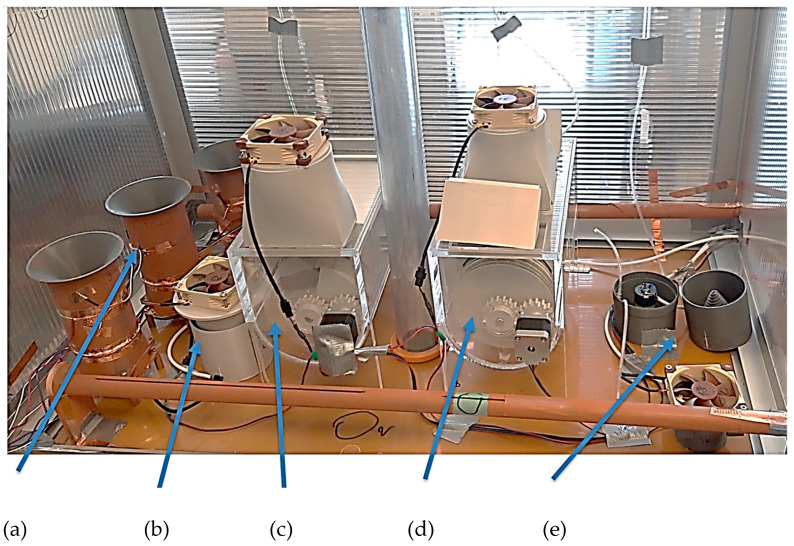
Test box with the sampling devices: (**a**) aerosol uses the electrodynamic separation of agents (EHDS): (**b**) large aerosol passage method (LAP) and direct contact with the preservative fluid; (**c**) gas-permeable mesh (GPM) setup and direct rinse with preservative fluid; (**d**) aerosol drift over (ADO) a large area washed with containment fluid; and (**e**) aerosol contact with liquid (ACWL) driven by a pump.

The devices tested under identical conditions can be characterized as the following:

(a) The device, which uses the electrodynamic properties of the agents for electrohydrodynamic separation of agents (EHDS) (Figure 8a), is based on the principle of separation of passing objects of the agents by Wien fitting and diverting the objects with a sowing electric charge to the designated prosample. The aerosol objects thus separated are collected in containers of preservative solution. The aerosol is carried away from the preceding structures by a fan, which creates a vacuum in the inlet of the device. This is to prevent any contact of the surface with the aerosol. The Wien filter consists of a magnetic part and an electrical part. The magnetic field has been set to a constant level *B*_avg_ = 0.2 T in the central part of the filter; permanent magnets are used, FeNeB, *H*_c_ = 850 kA/m, *B*_r_ = 1.2 T, and the electric field is set to a value *E*_1_ = 37.5 V/m, *E*_2_ = 150 V/m, and *E*_3_ = 750 V/m using the voltages set on the electrodes of the filter: *U*_w1_ = 1.5 V, *U*_w2_ = 6 V, *U*_w3_ = 30 V. EHDS has an active surface size *S*_act_ = 0.000400 m^2^ and the contents of the trapping fluid *V*_liq,act_ = 40 mL.

(b) The device using the large aerosol passage (LAP) method (Figure 8b) and having direct contact with the preservative liquid uses a set sensor cavity geometry solution to adjust the aerosol flow to repeatedly wash and contact the preservative solution level. A fan is placed in the inlet top of the device to draw in aerosol from the surrounding environment and, due to the shapes of the walls, create aerosol dynamics that repeatedly wash and contact the preservative solution level. The LAP has an active surface size *S*_act_= 0.00785 m^2^ and the contents of the trapping fluid *V*_liq,act_ = 50 mL.

(c) The device based on the gas-permeable mesh (GPM) method uses a direct aerosol rinse of a mesh carried on the surface of a cylinder (Figure 8c), which is placed with its axis horizontal in the lower position of the box rinsed with preservative liquid. The method uses a small amount of preservative liquid. When the net carrier is rotated while being rinsed with the aerosol blown into the box by the fan, the wetting of the net surface in the lower part of the tank is enhanced by the fact that the center, in the axis of the cylindrical surface of the net mounting, is a rising surface (the auger) which, when the net carrier is rotated, multiplies the contact surface of the net and the aerosol passing from the inside of the cylindrical surface (the net). GPM had an active surface size *S*_act_ = 0.141 m^2^ and the contents of the trapping fluid *V*_liq,act_ = 100 mL.

(d) A device using the aerosol drift method (ADO), which is based on setting up to 100 times the aerosol spray area in a snail-shaped aerosol with a small air gap, is shown in Figure 8d. The large area is then washed with a preservative solution. However, this has a larger volume relative to the GPM system because it must extend to the centerline of the cylindrical system’s cap in the tank. The incoming aerosol, blown in by a fan located at the top of the box, ensures aerosol contact on the preservative-wetted walls of the very dense starch wall, achieved by the small gap of the flowing aerosol. This achieves a multiple (100×) gain in aerosol contact area with the active preservative solution. ADO has an active surface size of *S*_act_ = 0.530 m^2^ and the content of the trapping fluid is *V*_liq,act_ = 200 mL.

(e) The device uses an aerosol contact with liquid (ACWL) (Figure 8e), such that the preservative solution driven by a peristaltic pump plasters a large surface area formed on a conical surface inside the device body. An aerosol is blown onto the conical surface by a fan. The large contact surface and the surface washing ensure multiple uses of the aerosol in contact with the preservative solution. ACWL had an active surface size of *S*_act_ = 0.00942 m^2^ and the content of the trapping fluid is *V*_liq,act_ = 40 mL.

**Figure 9:**
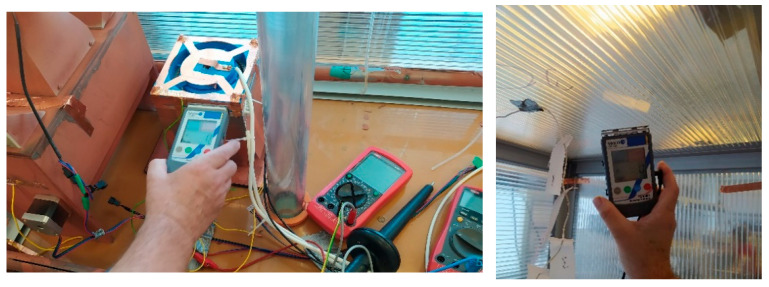
Test box with the sampling devices; electric charge test.

The validation experiment included the EHDS principle (Figure 8a) in addition to the wet samplers (LAP, GPM, ADO, ACWL). Aerosol was generated from a suspension containing 10^6^ FIPV viral particles and 10^8^ BS spores per mL. Samples of both model organisms were collected at 30 and 60 min intervals. This experiment was repeated with and without antistatic spraying of all instruments to assess the possible effect [13,15,17,22] of electrical charge on aerosol sampling. In this experiment, the inner walls of the enclosure were covered with an antistatic film, thus ridding the entire enclosure of electrical charge (Figure 9). The experiment involved the analysis of swabs from the inner surfaces [19,20,22,26,27].

**Figure 10:**
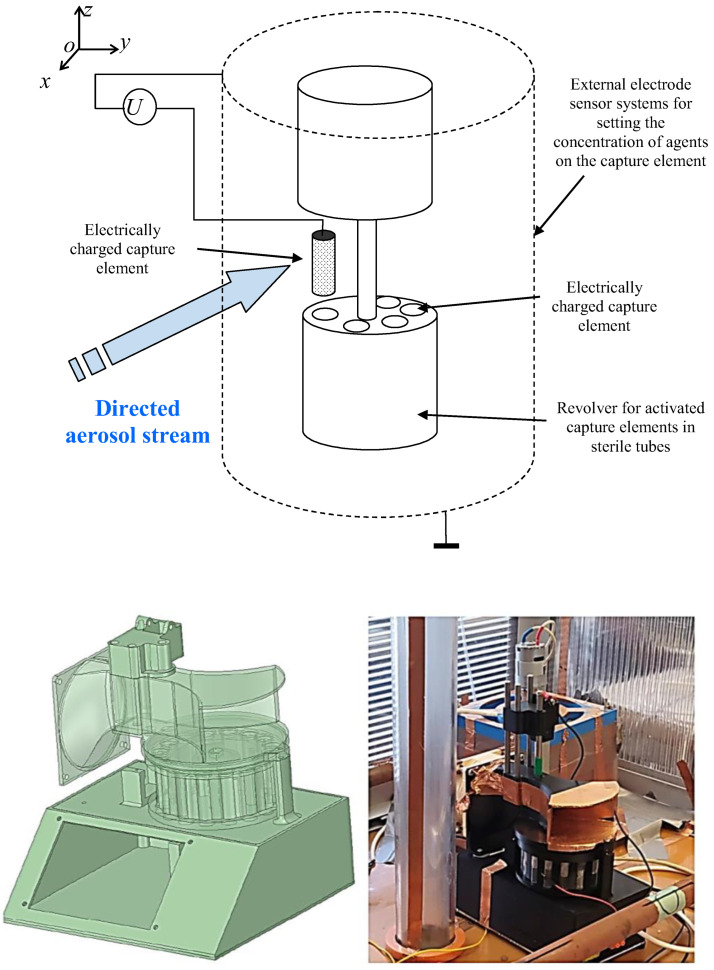
Device for semi-automated capture of FIPV and BS on the dielectric fabric of the r-EHDS system concept.

The construction of the sensor (Figure 10) was provided with an electric voltage source adjustable from 1 to 200 V for the necessary electric field intensity *E* created on the Wien filter electrode system [10,11,14,19]. This voltage was applied to the electrodes of the separator, and tests showed that the monitored virus aerosol had a non-zero resultant electrical charge. Furthermore, the capture site was made from a theoretical flat shape to a cylindrical one due to the possibility of semi-automatic exchange of polyethylene fabric capture samples (Figure 10).

The system automatically ejected the fabric from the skimmer and automatically re-inserted it after exposure to the aerosol. This method was advantageous for the safety of the laboratory staff. The device of the designed sensor with the electrodynamic principle of the agent concentration (Figure 10) was tested in several modes. In the first mode, biological objects—FIPV and BS—were actively scanned for *t* = 2 s, after which the capture module was replaced. Five simple capture modules were tested, as well as two sets of sample modules one to five under the conditions of an incoming aerosol with a concentration of 1.8·10^6^ cells/mL. The evaluation showed the repeatability of the measurements.

Using a 10 mL syringe, 10 mL of preservative liquid was aspirated at defined time intervals (wet sampling approach). Approximately 1 mL of liquid (preservative liquid) was placed in a 2 mL tube with a screw cap, and a wet collection sample was applied to it. The meshes from dry approach sampling were placed in 2 mL screw-cap tubes, and 600 μL of preservative solution was added. Additional swab samples (FLOQSwabs Genetics, Copan, Italy) taken from the surface of the instruments and from the surface of the inner walls of the cabinet were placed in 600 μL of preservation fluid in a 2 ml tube with a screw cap. Subsequent isolation of RNA and DNA was performed using the EliGene^®^ Viral DNA/RNA FAST Isolation Kit (ELISABETH PHARMACON, Czech Republic) according to the manufacturer’s procedures and recommendations. Evaluation techniques were based on previously published procedures [28].

**Detection and evaluation of the tested organisms by qPCR**

Detection and quantification of FIPV and B. subtilis were performed using primers and probes targeting the region of ORF1ab and gyrB, respectively. The qPCR assay was performed in 20 μL total volume using 500 μM of each primer, 250 μM probe, and 5 μL of isolated RNA/DNA.

The BS assay was performed in the same reaction volume with 1× EliZyme™ Probe MIX (ELISABETH PHARMACON) with 500 μM of each BS primer, 250 μM BS probe, and 5 μL. Both qPCRs were performed in a CFX96 instrument (Bio-Rad) under the following conditions:

- Reverse transcription was at 55 °C for 15 min.
- Initial denaturation was at 95 °C for 2 min and 45 cycles of 95 °C for 5 s, 55 °C for 15 s, and 67 °C for 15 s.

Each run included a 3-point calibration curve derived from the diluted qPCR product of FIVP and BS. Evaluation of results and quantification of FIPV and BS in isolated RNA/DNA were performed in Bio-Rad CFX Maestro 2.3 software.

The tests specified above under the conditions from Table 1 were repeated several times (5×). The repeatability and detection capabilities of the tested virus- and bacteria-sensing systems based on the principle of electrohydrodynamic sensing agents were demonstrated. According to the obtained results (Figure 10), the validity of the proposed and implemented methods of capturing selected biological objects was proven.

## 3. Design of a Fully Automated Agent Collection System

Comparative tests of the designed and tested virus and bacteria sensors (Figure 8) and comparisons using swabs at different locations of the aerosol box and electrical charge monitoring gave a clear answer that the electrical charge effect is the dominant factor in the success and sensitivity of agent capture.

**Figure 11:**
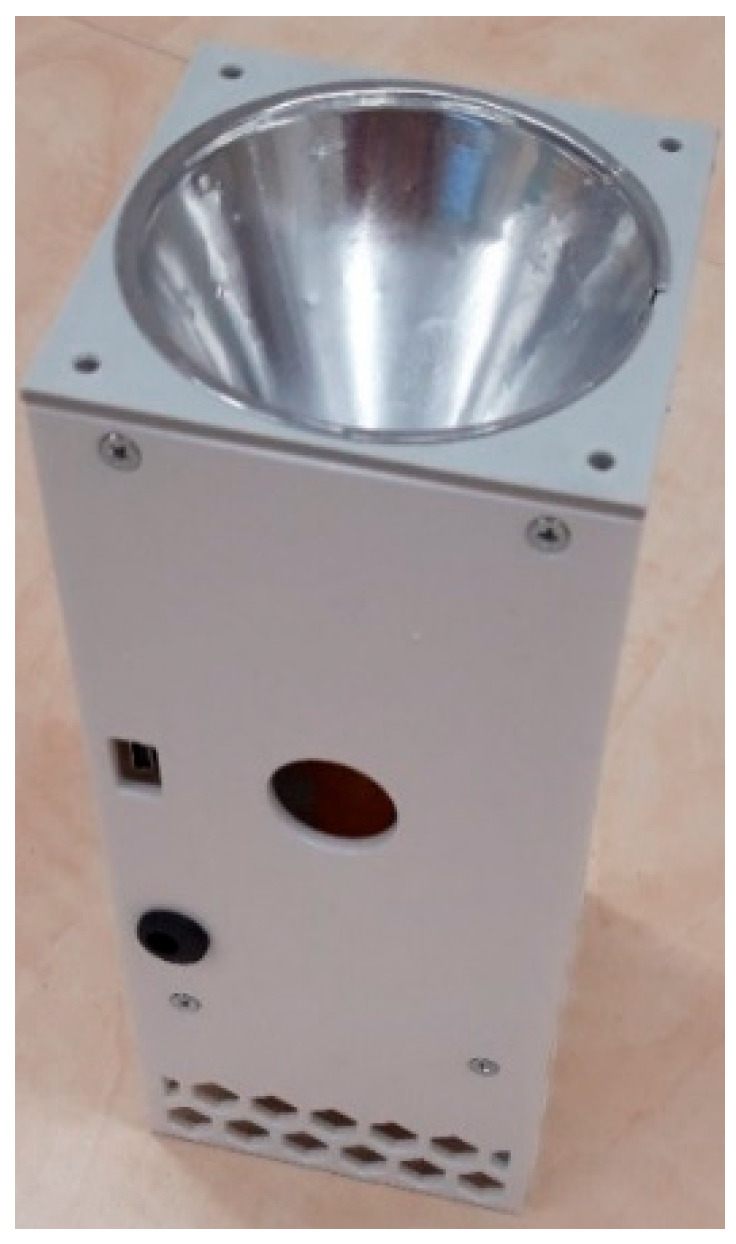
Final functional device for fully automated FIPV and BS capture on r-EHDS dielectric fabric.

From the design of Figure 10, a design direction of a simple sensing mobile device (Figure 11) emerged that satisfies all requirements. It was tested for quality, sensitivity, and reliability of agent capture. The tests differed in the set electrical intensity of the electrode system in the fabric area as well as the time of intensity setting. After several systematic tests, the following parameters were found to be the best: *E*= 480 V/m, *t*_exp_ = 60–90 min.

**Figure 12:**
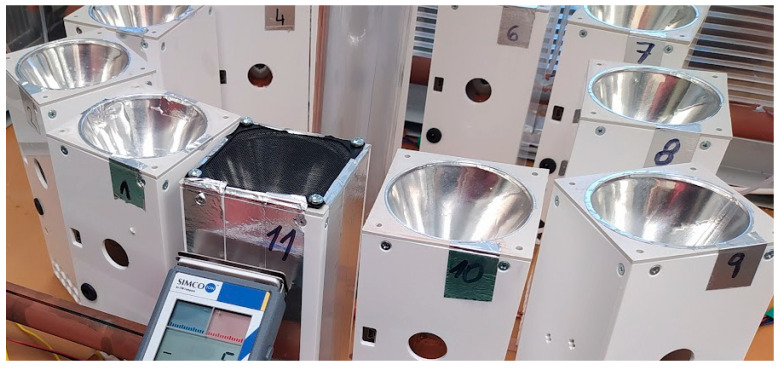
Laboratory test of a fully automated agent trapping device on an r-EHDS dielectric fabric.

The 11 pieces of devices assembled in this way were tested under the same conditions in an aerosol box with FIPv and BS (Figure 12). After this laboratory test, the sensors were deployed in real-world environments in real shopping malls and student canteens.

## 4. Evaluation of FIPV and BS Capture by the Designed Sensor

The design and subsequent construction of the final sensing devices (Figure 10, Figure 11 and Figure 12) in the laboratory tests of the aerosol units with FIPV and BS agents located in the test chamber in the laboratories in Těchonín show the values listed in Table 2, Table 3 and Table 4. An aerosol of a defined concentration (FIPV concentration: 1.8·10^6^ cells/mL) was applied, and then the recovery was evaluated by laboratory PCR assays. For curves above the threshold of PCR detection, the data for sensors are tabulated.

A test was also conducted with varying concentrations of FIPV (1.2·10^4^ cells/mL, 1.8·10^5^ cells/mL, 1.8·10^6^ cells/mL) in the fogger. The results of the baseband detection are shown in Table 5. The proposed system increases its capture efficiency in proportion to the intensity of the electric field E at the capture matrix. To decouple detection efficiency from *E* (since pathogens have field thresholds that may break down their DNA into undetectable fragments for PCR), time exposure of the capture matrix was tested in the range of 30 s to 120 min. Results showed that under a sub-threshold electric intensity *E*_crit_, similar outcomes were achieved with longer exposure compared to shorter exposure at the threshold electric intensity level. A more detailed statistical evaluation of the results of the first measurement with a reading of *N* = 11 samples is statistically evaluated in Table 6.

The final sensor design respects the influence of a charged object (e.g., FIPV, BS microorganism) within a controlled electric field. Existing particle separation devices (e.g., for air pollution control in energy and marine industries) use the maximum achievable electric intensity *E* to ensure particle capture. However, in our case, exceeding a critical electric intensity *E*_crit_—specific for each microorganism—results in DNA degradation and pathogen disintegration. Thus, PCR can no longer confirm its presence. These devices are therefore unsuitable for microbiological sampling where DNA detection is required.

Other devices, such as electrostatic aerosol samplers for optical and electron microscopy [29,30], operate under entirely different pressure and aerosol concentration conditions compared to our system, which works with ambient air. The design presented in [31], “A prototype personal aerosol sampler based on electrostatic precipitation and electrowetting-on-dielectric actuation of droplets,” appears superficially similar, but it applies +/−4.7 kV electrode voltages. Given its construction and electrode potential, it likely destroys biological particles and DNA, rendering it unsuitable for pathogen sampling and detection.

The pathogen capture device (see Figure 12) is restricted to non-liquid environments. The tested minimum capture distance is d = 2 m. Special tests for detection sensitivity at greater distances may be added in future research. The device uses a microcontroller and a Li-Pol battery that enables 24 h operation according to preset programs. The system is not significantly affected by external electric fields.

The processing of the measured results was evaluated for type A uncertainty. The mean value *x*_AVG_ of the cycle in which 1000 RFU of the PCR method was exceeded and its scatter for the compared measurements of the tested different basic concentrations in the fogger were statistically evaluated. The statistical evaluation of the uncertainty for the magnitude of the mean value *x*_AVG_ and its scatter *x*_scat_ for *N* samples of the selected concentration was performed according to the relationships:

(3) $$x_{\mathrm{AVG}}=\frac{1}{N_{test}}\sum_{i=1}^{N_{test}}\left(x_{i}\right),$$

(4) $$x_{\mathrm{scat}}=\sqrt{\frac{1}{N_{test}-1}\sum_{i=1}^{N_{test}}{\left(x_{i-x_{AVG}}\right)}^{2}}$$

## 5. Conclusions

The proposed and described new method of separation and capture of agents (FIPV and BS) for subsequent evaluation of their presence in aerosol using PCR tests has proven unambiguous capture of these specified biological objects both in laboratory tests and in the real environment. This is a completely unique system, not implemented anywhere in 2021–2023. The method is also suitable for sensing other nano-objects of biological nature from aerosol in the air with a reported non-zero electric charge. A validation procedure and design were theoretically developed, and the proposed model was numerically analyzed and evaluated. The subsequent design of structures as a technical solution for the capture system was embodied in a functional device using a disposable, fully automatic rapid prototype sensing device. It was first tested and verified in the laboratories of UTEE FEKT BUT Brno to confirm the electromagnetic parameters and then in the control boxes of the Těchonín CZ biolaboratory.

Based on this procedure, a functional automatic sensor for capturing and evaluating agents in areas with significant population density was designed, which was realistically tested outside the laboratory, and the detection yielded positive results (7 positive SARS-CoV-2 out of 10 deployed in public rooms or areas). It was shown that the electromagnetic and electrodynamic object separation system has significant positive properties and advantages in the quality and sensitivity of detection compared to the mechanistic–fluidic methods used so far.

The advantage of the proposed automated aerosol pathogen capture method combined with PCR analysis, compared to traditional surface swabbing and PCR, is the automated and controlled sampling of aerosols in nearly any defined airspace. GPS location data can be added, and personnel requirements reduced to only once every 24 h or as needed across an urban area. Manual swabbing and PCR analysis are labor- and expertise-intensive and may not offer the same metrological validity as automated, standardized capture.

## Figures and Tables

**Table 1:** Comparison of intercepts for the proposed tests *t* = 30 min of exposure, number of repetitions *N*_t_ = 5.

Method	Active Surface *S*_act_ [m^2^]	Concentrate FIPV [Cells/mL]	Detection (Cycle Number When Exceeded 1·10^3^RFU PCR Method)
**LAP**	0.00785	1.8·10^6^	36
**GPM**	0.141	1.8·10^6^	34
**ADO**	0.530	1.8·10^6^	34
**ACWL**	0.00942	1.8·10^6^	36
**EHDS**	0.000400	1.8·10^6^	36
**Comparative sampling**	Sludge-contact	1.8·10^6^	26

**Table 2:** Measurement results for the proposed EHDS solutions: *t*_elements_ = 2 min. exposure; number of repetitions of the test *N*_test_ = 30; electric intensity electrode sensor system *E* = 288 V/m.

Method	Active Surface Sact [m^2^]	Concentrate FIPV [Cells/mL]	Detection (Cycle Number When Exceeded 1·10^3^RFU PCR Method)
**EHDS**	0.000314	1.8·10^6^	36 ± 1.5
**Comparative sampling**	Sludge-contact	1.8·10^6^	28

**Table 3:** Measurement results for the proposed r-EHDS solutions: telements = 2 min. exposure; number of repetitions of the test *N*_test_ = 11; electric intensity electrode sensor system *E* = 288 V/m.

Method	Active Surface Sact [m^2^]	Concentrate FIPV [Cells/mL]	Detection (Cycle Number When Exceeded 1·10^3^RFU PCR Method)
**r-EHDS**	0.000314	1.6·10^5^	36 + 2
**Comparative sampling**	Sludge-contact	1.6·10^5^	32

**Table 4:** Measurement results for the proposed r-EHDS solutions: *t*_elements_ = 60 min. exposure; number of repetitions of the test *N*_test_ = 11; electric intensity electrode sensor system *E* = 480 V/m.

Method	Active Surface Sact [m^2^]	Concentrate FIPV [Cells/mL]	Detection (Cycle Number When Exceeded 1.10^3^RFU PCR Method)
**r-EHDS**	0.000314	1.8·10^6^	26 + 2
**Comparative sampling**	Sludge-contact	1.8·10^6^	22

**Table 5:** Results of approximate measurement evaluations for the proposed r-EHDS solutions: *t*_elements_ = 60 min. exposure; number of repetitions of the test *N*_test_ = 11; electric intensity *E* = 288 V/m for different concentration agents.

Method	Active Surface Sact [m^2^]	Concentrate FIPV [Cells/mL]	Detection (Cycle Number When Exceeded 1·10^3^RFU PCR Method)
**r-EHDS**	0.000314	1.2·10^4^	38 + 4
**Comparative sampling**	Sludge-contact	1.2·10^4^	36
**r-EHDS**	0.000314	1.8·10^5^	34 + 2
**Comparative sampling**	Sludge-contact	1.8·10^5^	32
**r-EHDS**	0.000314	1.8·10^6^	26 + 2
**Comparative sampling**	Sludge-contact	1.8·10^6^	22

**Table 6:** Results of measurement evaluation with regard to agent capture statistics at different concentrations: r-EHDS solutions, *t*_elements_ = 60 min. exposure; number of repetitions of the test *N*_test_ = 11; electric intensity *E* = 288 V/m for different concentrations of FIPV.

Method	Active Surface Sact [m^2^]	Concentrate FIPV [Cells/mL]	Average Detection Value (Cycle Number When Exceeded 1·10^3^RFU PCR Method), *x*_AVG_	Scatter Value (Cycle Number When Exceeded 1·10^3^RFU PCR method), *x*_scat_
**r-EHDS**	0.000314	1.2·10^4^	38.60 *	1.87 *
	Sludge-contact	1.2·10^4^	35.81	1.53
**r-EHDS**	0.000314	1.8·10^5^	34.90	1.81
	Sludge-contact	1.8·10^5^	32.72	1.79
**r-EHDS**	0.000314	1.8·10^6^	26.09	1.70
	Sludge-contact	1.8·10^6^	22.18	1.32

* *N*_test_ = 9 valid samples were evaluated.

## Data Availability

The original contributions presented in this study are included in the article. Further inquiries can be directed to the corresponding author(s).
